# Biological function in the twilight zone of sequence conservation

**DOI:** 10.1186/s12915-017-0411-5

**Published:** 2017-08-16

**Authors:** Chris P. Ponting

**Affiliations:** MRC Human Genetics Unit, The Institute of Genetics and Molecular Medicine, University of Edinburgh, Western General Hospital, Crewe Road, Edinburgh, EH4 2XU UK

## Abstract

Strong DNA conservation among divergent species is an indicator of enduring functionality. With weaker sequence conservation we enter a vast ‘twilight zone’ in which sequence subject to transient or lower constraint cannot be distinguished easily from neutrally evolving, non-functional sequence. Twilight zone functional sequence is illuminated instead by principles of selective constraint and positive selection using genomic data acquired from within a species’ population. Application of these principles reveals that despite being biochemically active, most twilight zone sequence is not functional.

## Function versus conservation versus constraint

Functionality of most human protein coding, and some non-coding, sequence is clearly implied when it is conserved across diverse mammalian species. This has been a rule-of-thumb by which to infer whether a sequence is functional without the benefit of experimental data. Conservation, however, is not a faithful indicator of functionality. High sequence conservation could reflect a relatively brief period of neutral evolution over which few mutations accumulated. Just because approximately 98% of human DNA is conserved in chimpanzee, for example, this does not imply that this amount of sequence conveys function. Conversely, poor conservation of a sequence does not imply that it is devoid of function. After all, low conservation could also be explained by frequent episodes when rare mutations are brought to high frequency and fixation within a population by positive selection. Thresholding on percentage nucleotide sequence identity thus fails to neatly separate functional from non-functional sequence. This means that as sequence conservation diminishes we drop into a ‘twilight zone’ [1, 2] in which DNA cannot immediately be ascribed as either functional or non-functional. Population genetics principles illuminate the functionality of sequence in the twilight zone. These can be used to assess whether sequence evolution has been constrained, meaning that it exhibits a slower rate of change than predicted by a model of neutral evolution; selective constraint is inferred by considering the degree by which allele frequencies are depressed across extant populations [3–5]. Conversely, functional sequence subject to positive selection exhibits a rate of change greater than seen for neutrally evolving sequence.

Sequence conservation and constraint are not the only benchmark by which to evaluate functionality. High throughput experimental assays are providing genome-wide assessments of functional sequence. Armed with this experimental information, can we now reveal the extent of functional sequence and associated molecular and cellular biology present in the twilight zone of low sequence conservation? Here I review instances where sequence is functional despite its low conservation, focusing principally on our own and other mammalian species. I conclude that population genomics-based approaches to predict function are paramount because, counterintuitively, experiments are not perfect predictors of function.

## A twilight zone protein-coding gene

**Fig. 1.**
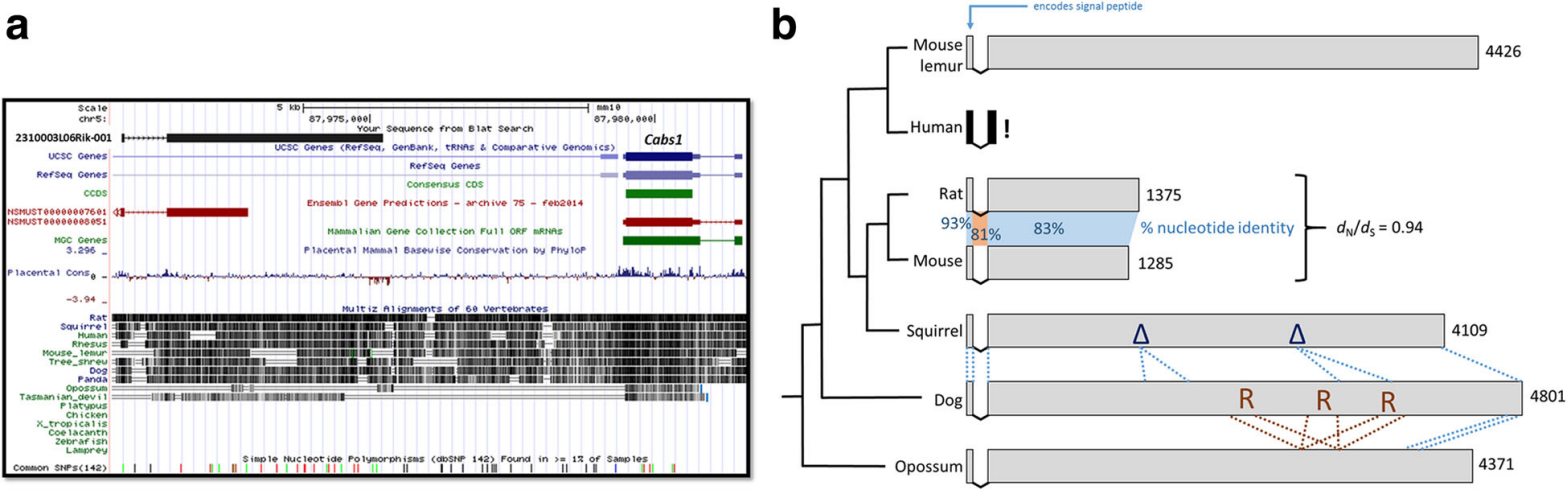
Rapid evolution among 2310003L06Rik orthologues. **a** Nucleotide conservation is low across placental mammals, and the locus is not aligned with non-mammalian species. The mouse gene is incompletely predicted in Ensembl, and absent from other databases such as RefSeq and CCDS. **b** Rapid evolution of mammalian 2310003L06Rik orthologues. Open reading frames (shown in *grey*) are of highly variable length (amino acid numbers shown on the *right*) across mammalian species (phylogeny shown on *left*, not to scale). Human, chimpanzee and macaque genomes contain nucleotide substitutions that truncate the open-reading frame (“*!*”; pseudogene indicated in *black*). Deletions in the squirrel (*Spermophilus tridecemlineatus*) orthologue, relative to the dog, are indicated by “Δ”, and repeats in the dog sequence are shown by “*R*”. Aligned protein sequences are indicated by *dotted blue* and *brown lines*. There is no significant sequence similarity evident between mouse and opossum (*Monodelphis domestica*) orthologues (blastp *E* > 0.2). *d*_*N*_ */d*_*S*_ is explained in the legend to Fig. 2

The 2310003L06Rik gene exemplifies the rapidity with which a locus can evolve (Fig. 1). Little is known about its function, except that in mouse gene expression is specific to the tongue. With regards to evolution, it is a member of the secretory calcium-binding phosphoprotein (SCPP) gene family [6, 7] located in a tandem array on mouse chromosome 5, including those encoding enamel matrix proteins, milk caseins and salivary proteins, which mostly arose by local gene duplication and subsequent divergence during early mammalian evolution. In four respects, this gene is not well conserved: (i) it is present only in theria (marsupials and placental mammals) but not in monotremes; (ii) its amino acid sequence varies greatly, with a 3.7-fold difference in length between mouse and dog; (iii) it contains lineage-specific repeats and insertions or deletions; and (iv) in some lineages, such as the Catarrhini (including human), it has acquired open-reading frame disruptions and thus has become a pseudogene. Nucleotide sequence similarities between closely related species, such as mouse and rat, differ little between exons and introns and its protein sequence has evolved at a rate near to that of synonymous sites, often used as a neutral rate proxy. Of all its many features, conservation is evident only in these orthologues’ initiating codon, their common number of exons and their splice sites.

**Fig. 2.**
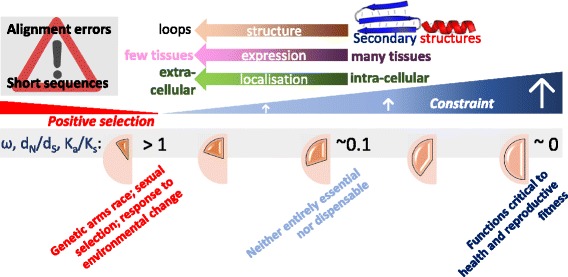
Trends regarding sequence constraint. Protein-coding sites that are highly constrained (*d*_*N*_ */d*_*S*_ → 0) tend to fall within secondary structures within intracellular proteins expressed in many tissues, whereas the less-numerous sites that are evolving either near neutrally (*d*_*N*_ */d*_*S*_ ≈ 1) or in response to positive selection (*d*_*N*_ */d*_*S*_ > 1) tend to lie in disordered regions or in loops in secreted proteins that are expressed in a tissue-restricted manner [73–75]. The median value of *d*_*N*_ */d*_*S*_ for human and mouse orthologues is 0.095 [76]. Inferences of positive selection (for example using PAML [77]) can be in error due to sequence misalignment [78, 79], or when alignments are short [80], or when *d*_*N*_ exceeds *d*_*S*_ because of chance fluctuations. *d*_*N*_ */d*_*S*_ (also written as *K*_*a*_ */K*_*s*_ or ω) [81] is the ratio of the number of nonsynonymous substitutions per nonsynonymous site (*d*_*N*_) to the number of synonymous substitutions per synonymous site (*d*_*S*_)

Functional sites that are neither well conserved nor constrained fall into two classes that differ in the rate by which they accumulate mutations relative to the neutral rate (Fig. 2). Sites in the first class evolve rapidly due to positive selection and adaptation. This is when rare mutations confer reproductive advantage leading to their rise in frequency and their fixation in that population faster than neutral mutations. In mammals, most positively selected substitution events occur outside of DNA that encodes protein [8]. Nevertheless, they are particularly concentrated in the ~1% of genomic sequence that is protein-coding and their density is low overall in the non-protein-coding portion [8]. The second class of functional yet poorly conserved sequence evolves by weak negative selection [9]. Such sites accumulate substitutions, on average, slower than the neutral rate and show a low degree of constraint. Variants at these sites that have only a slight deleterious effect on fitness can become fixed in populations, which is a consequence of natural selection being unable to discriminate effectively between slightly deleterious and neutral mutations [3, 10] (discussed below).

Has a poorly conserved homologous sequence diverged by weak negative selection or else by positive selection? Answering this question computationally remains a substantial challenge because some approaches are associated with high rates of false positive predictions [11]. The most compelling examples are when candidate positively selected sites are spatially clustered within ligand-binding pockets, such as observed in mouse major urinary proteins [12] or in major histocompatibility complex class I subunits [13]. As with these two studies, clear-cut instances are often found for proteins involved in reproduction—because of the genetic arms race inherent in sexual selection [14]—or in immunity and host defence [15]. The genetic arms race with viruses, in particular, is predicted to account for nearly a third of all positively selected change occurring in human protein sequence that is conserved across mammals [16]. The evolution of primate and bat poly-ADP-ribose polymerases, for example, appears to have been subject to considerable genetic arms races with unidentified pathogens, resulting in positively selected sites that cluster in three dimensions and in a disordered region of unknown function [17].

Genes whose variants have been positively selected, including those involved in reproduction and host defence, are often in large families whose numbers are not well conserved between, or even within, species owing to high rates of duplication and/or pseudogenisation [18–20]. Nevertheless, basal mutational rates of duplication and loss are highly variable; hence, in most cases it is difficult to evaluate the contribution made by selection in retaining or purging gene duplicate and gene disruptive alleles in the population [21]. Some examples in human evolution are more compelling because of their ability to link copy number variation with fitness. A higher gene copy number of *CCL3L1*, which encodes a known ligand for the human immunodeficiency virus (HIV) co-receptor CCR5, for example, is associated with lower susceptibility to HIV and to acquired immunodeficiency syndrome, and even higher copy numbers are observed in chimpanzees [22]. In general, however, despite their high prevalence, with four-times more human nucleotides present in copy number increased regions than in single nucleotide variant sites, copy number gain of human genes appears to be under little or no selection [23].

To summarise the hallmarks of a rapidly evolving gene, I return to the 2310003L06Rik protein-coding locus (Fig. 1). It is a member of a large multi-gene family (namely SCPP genes) whose genes duplicated and became pseudogenes rapidly over mammalian evolution; it encodes a secreted protein, which means perhaps that it is more likely to be engaged in inter-specific conflict between host and pathogens; this protein’s structure is apparently flexible and disordered, which is less likely to evolve by purifying selection; and, its expression profile is narrowly restricted to few tissues, indeed to only one, the tongue. Nevertheless, in the absence of statistical evidence that this gene has experienced episodes of positive selection, it need only be stated that its evolution has been more rapid than that of most mammalian genes.

## Twilight zone non-protein coding genes

**Fig. 3.**
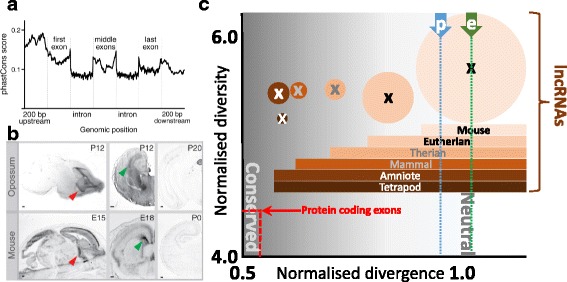
Most mouse lncRNAs are not conserved in sequence or in transcription. **a** Pan-vertebrate conservation is low in promoters and exon sequence, as indicated, for a generic lncRNA locus. Y-axis: conservation (phastCons) scores sampled across 877 mouse multi-exon lncRNAs (reproduced from [82]). **b** An exception that establishes a more general rule: expression conservation of a lncRNA (AK082072) across mouse and marsupial (*Monodelphis domestica*) brain development (reproduced from [82]). **c** Normalised divergence (*X-axis*; *d/d*_*AR*_) and diversity (*Y-axis*; *π/d*) of lncRNAs whose expression is restricted to mouse, eutheria, theria, mammalia, amniota and tetrapoda where the *area of a circle* indicates relative lncRNA numbers (7306 in the mouse-only set). *Divergence* is the mouse-rat median substitution rate normalised by the local neutral mutation rate; *diversity* is the mouse median nucleotide diversity divided by local mouse-rat divergence. Increasing conservation is indicated by darker background hues. For comparison, significantly reduced divergence and diversity values are evident for protein-coding exons (shown in *red*); in general, tissue-specific transcription of protein-coding orthologues is highly conserved [83]. The diversity (*π/d*) of only eutherian-specific lncRNAs differs significantly from proposed neutral sequence. For a description of the data, definitions of *d, d*_*AR*_ and *π*, and further details see [32]. Median *d/d*_*AR*_ divergence estimates of promoter-like (p) and enhancer-like (e) lncRNA exons [33] are indicated by *vertical blue* and *green dotted lines*, respectively

If we conceive of a spectrum of conservation with most protein-coding genes placed at one extreme because of their strong degree of constraint, then long non-coding RNAs (lncRNAs) are located at the other: most multiexonic lncRNA loci exhibit little or no cross-vertebrate sequence conservation (Fig. 3) [24]. Where conservation exists it need not reflect an RNA-mediated function, but could be explained instead by functional elements contained within the underlying DNA that are crucial to the normal function of an adjacent protein-coding gene. Nevertheless, a degree of constraint on lncRNA transcription and splicing is evident within lncRNA promoters, splice sites and exons [25–28]. In contrast to most mammalian protein-coding genes, which possess homologues that are identifiable across diverse animal phyla and beyond, 80% of human lncRNA families originated recently during primate evolution, and only 3% are conserved in more distantly related species such as chicken or frog [29, 30]. Not only conservation, but also constraint and positive selection, are low or absent on intergenic lncRNAs among modern human or mouse populations [29, 31, 32].

lncRNAs are considered to fall into two distinct classes: enhancer-like lncRNAs show no sequence conservation, whereas promoter-like lncRNA exons are modestly conserved (Fig. 3) [33]. Promoter-like lncRNAs are thus the more likely to possess RNA sequence-dependent functions. The more numerous enhancer-like lncRNAs also show poorly conserved transcription, and likely contribute many of the 40% of mouse loci whose transcription fails to be conserved in the rat in the same tissue [34]. In the absence of frequent sequence and transcriptional conservation, and until there is experimental evidence of RNA-dependent function, such enhancer-like lncRNAs will not justify consideration as genes. For promoter-like lncRNAs, RNA sequence-dependent function could be mediated by secondary structure. Nevertheless, there is no support for proposed conserved secondary structures of well-studied lncRNAs, such as *HOTAIR*, *SRA*, and *Xist*, from pairwise covariation in sequence changes [35].

Shorter (~22 nucleotide) microRNAs are also often lineage-specific [36]. Placental and marsupial mammals have experienced a net gain of nearly one new microRNA family per million years, over twice the rate observed in birds [37]. Once a new family arises, it can expand rapidly by tandem duplication and lose members by pseudogenisation, as observed for a primate-specific family of 46 microRNAs present on human chromosome 19 [38]. Concomitantly, mRNA targets of these microRNAs can evolve by the gain or loss of binding sites within mRNAs’ 3′ UTRs [39, 40].

**Fig. 4.**
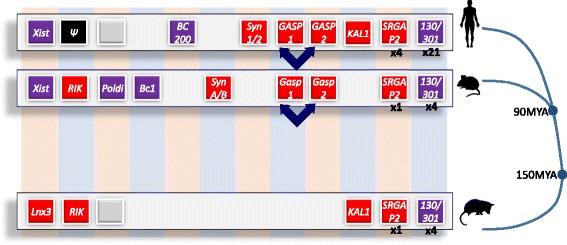
Lineage-specific genes arise through gene birth, death and duplication and exaption of transposable elements. Examples of lineage-specific protein-coding genes (shown in *red*) or non-coding genes (shown in *purple*) present in one or more of the human, mouse and opossum (*Monodelphis domestica*) genomes. Orthologous genes are indicated across vertical columns. Where orthologous sequence is absent, due to a lineage-specific insertion or deletion, no boxes are shown. The eutherian *Xist* noncoding RNA gene arose, in part, from disruption of an ancestral *Lnx3* protein-coding gene [84]. The 2310003L06Rik gene (‘RIK’) is disrupted in human (Fig. 1). The *Poldi* ncRNA gene arose de novo, within the last ~3.5 million years, in the mouse lineage within untranscribed sequence [85]. *Grey boxes* indicate non-genic sequence in human and in opossum that is orthologous to the mouse *Poldi* locus but that has no conservation of transcription. Rodent *BC1* and primate *BC200* noncoding genes arose independently from separate retrotransposition events yet bind the same protein, FMRP [86]. Similarly, syncytins 1 and 2 arose from endogenous retroviral element insertions in the primate lineage, and separately syncytins A and B arose from such insertions in the rodent lineage [87]. *Dark blue double-headed arrows* indicate lineage-specific episodes of gene conversion between the two 5′ UTRs of *GASP1* and *GASP2*, genes that are placental mammal-specific [88]. The *KAL1* (anosmin-1) gene is entirely absent, and inferred to have been deleted, from the mouse genome [76]. Three duplications of human-specific *SRGAP2A/B/C* genes occurred within the last approximately 3.4 million years [89]. Four members of the microRNA 130/301 family are present in both the opossum and mouse genome, but 21 members are found in the human genome [36, 90]. *MYA* million years ago

Figure 4 summarises the preceding two sections on lineage-specific genes using examples drawn from gene birth, death, transformation and conversion, focusing specifically on the human genome.

## Non-conservation of the non-functional genome

Evolution of the mammalian genome is dominated not by conservation and stasis but by tumult and large-scale change [41]. The human genome, for example, is estimated to have lost 22% (700 Mb) of its DNA and gained an equivalent amount over the last 75 million years [42]. Chromosomal gene content—even between closely related species—is rarely conserved. An extreme example of this is the genomes of Indian and Chinese muntjak deer that have dramatically differing numbers of chromosomes (6 and 46, respectively) despite sharing a common ancestor within the last 2 million years [43].

Most non-conserved sequence lies within the non-functional ~92% of the mammalian genome [4, 44]. Rapid resculpting of mammalian genomes is dominated by lineage-specific insertion and deletion of transposable element (TE) sequence whose debris, together with other repetitive sequence, contribute up to two-thirds of the human genome [45]. Although occasionally it is proposed that a large fraction of TEs are functional [46], there is no evolutionary or experimental evidence to support this. Conversely, because the locations of insertion or deletion mutations in TEs occur almost exactly as would be expected from random events, the vast majority of TEs appear to be inert [47], with less than 2% of TE sequence (approximately 20 Mb) bearing the signature of constraint [44, 48]. The exceptions are, nevertheless, of interest: for example, 18 human *Alu* elements have evidence for being translated [49]; a handful of syncytin protein-coding genes have their origins in TEs (Fig. 4); and several families of microRNAs have derived from TEs, albeit slowly over evolutionary time [50, 51].

## Twilight zone of the functional genome

**Fig. 5.**
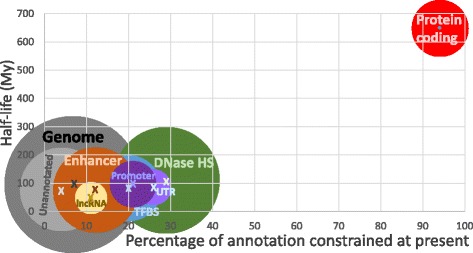
Protein-coding sequence turns over very slowly but is a minority of all constrained sequence; functional non-coding sequence turns over rapidly and contributes most to constrained sequence. *Circle areas* reflect proportions of annotations that are constrained (~8% for the mammalian genome); annotations are not mutually exclusive. Protein-coding sequence half-life is approximately the age of bilateral animals, and the half-lives of functional non-coding sequence are approximately the age of the radiation of placental mammals [91–93]. Further details of the data and the evolutionary model are provided in [44]

The ~8% functional genome, however, has also been altered greatly over tens of millions of years of mammalian evolution, with slow and fast rates of change for the functional protein-coding and functional non-coding portions of the genome, respectively (Fig. 5). Two extant species that last shared a common ancestor near the emergence of bilateral animals, 650 million years ago, are estimated now to share only half of their constrained protein-coding sequence. The equivalent half-life for functional non-coding sequence is considerably shorter, at approximately 75–100 million years [44] (Fig. 5).

Open chromatin, which contains many protein-binding sites, contributes both the largest amount of functional (area of circles in Fig. 5) and the greatest density (Fig. 5, X-axis) of functional sequence. Nevertheless, such sites, and promoters and enhancers more generally, are poorly conserved across mammals [52–55]. It is estimated that promoters for over 40% of genes have arisen or been lost in either the human or mouse lineage since their last common ancestor [55]. In a comparison of human, mouse, dog, opossum and chicken, most binding events were unique to one of these species [53]. In large part, the rapidity by which proteins’ DNA binding sites are gained and lost is explained by their short length. In a 1-kb segment of human DNA it is predicted that a new 7–8 bp protein-binding motif arises, by neutral evolution, on average every 60,000 years [56].

## Non-adaptive explanations of rapid evolution

**Fig. 6.**
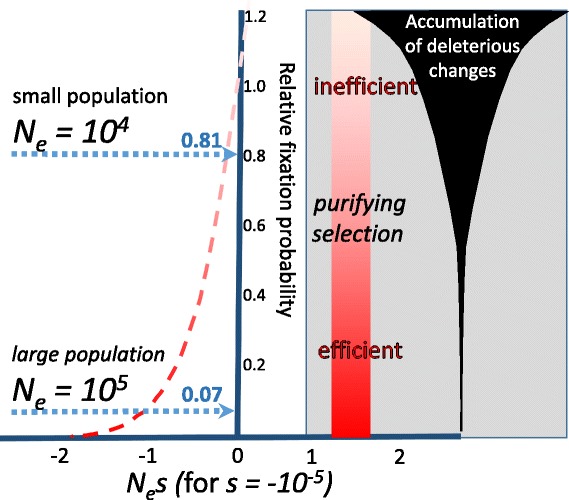
Variation in selection efficiency. Purifying selection is increasingly inefficient for alleles of small selection coefficient *s* within species of relatively small effective population sizes *N*_*e*_, leading to an increasing rate of accumulation of deleterious changes. The graph shows the probability of fixation of a new variant relative to the neutral expectation (*Y-axis*) as a function of *N*_*e*_ *s* for *s* = −10^−5^ (modified from [10]). For larger values, such as *N*_*e*_ = 10^5^, the probability of fixation relative to the neutral expectation is small at approximately 7%. Nevertheless, in a population with a tenfold smaller *N*_*e*_ this probability rises to 81%

Turnover of functional sequence, and allelic changes in gene repertoire, do not need to improve reproductive fitness. Instead, many changes have been deleterious, yet have not been removed by negative selection (reviewed in [10, 57]). In particular, alleles that have only a modest negative effect on fitness (small negative selection coefficient, *s*) will only have a strong likelihood of being purged from a species when its effective population size (*N*_*e*_) is large (Fig. 6). Conversely, when *N*_*e*_ is small, as it is for modern humans, then weakly deleterious variants show a greater chance of becoming retained. This implies that many variants that disrupt or delete genes, especially those with only subtle changes to organismal phenotype, will have been fixed despite being deleterious.

Rapid evolution could also reflect higher than average mutation rates. Sequence with a high CpG dinucleotide content, including protein-coding sequence, evolves particularly rapidly owing to a high rate of mutation from the methylated form of CpG to TpG and CpA in germline genomes [58–60]. Sequence lying within the highly recombining regions of the genome also evolves especially rapidly, with one mouse gene experiencing a 100-fold increase due to this phenomenon of biased gene conversion [61, 62]. Functional regions of the non-coding genome can also mutate rapidly due to DNA-bound factors blocking the displacement of error-prone polymerase-α sequence during replication [63]. Identifying sequences under positive selection due to adaptation is thus made more complex because not just the classical neutral model, but also models accounting for these mutational biases, need to be rejected.

## Concluding remarks: what do we mean by function?

On one hand, 80% of the human genome has been annotated by experiment either as being bound by proteins or as being the substrate of enzymatic activity, the majority of which overlaps with the ~67% of the genome that is TE-derived. On the other hand, this is far more than the ~8% of the human genome that shows evolutionary evidence of constraint, and there is evidence that only very few TEs aligned between species’ genomes are constrained (see above). Resolution of this apparent paradox stems from the realisation that many (even the majority of) molecular phenomena in cells are inconsequential in the sense that they are not surveyed effectively by natural selection [64, 65]. These phenomena include non-functional RNA–protein or protein–protein or protein–DNA interactions [66–68]. In the latter case, most interactions between proteins and chromatin have been shown as failing to alter transcription of putative target genes [68]. Current experiments are thus unable to distinguish cleanly between molecular activities that are incidental and those that are consequential, even vital. By contrast, evolutionary approaches can infer function, annotating sequence by the importance attributed to it by natural selection. Whilst problems remain to be overcome [69, 70], such approaches can discern lineage-specific function in sequence that is not conserved among species, and the absence of function in aligned, notionally conserved sequence [3]. Human genome sequencing at the population level is now accelerating [5, 71, 72]. The resulting extensive diversity data will permit the inference of constraint at high resolution and will thus shed light on function and molecular mechanisms. It will also help to overthrow misguided notions that function requires between-species sequence conservation or that function is widespread outside constrained sequence.
